# Response: Commentary: “*Prdm13* regulates subtype specification of retinal amacrine interneurons and modulates visual sensitivity”

**DOI:** 10.3389/fncel.2015.00520

**Published:** 2016-01-28

**Authors:** Yuko Sugita, Satoshi Watanabe, Takahisa Furukawa

**Affiliations:** Laboratory for Molecular and Developmental Biology, Institute for Protein Research, Osaka UniversityOsaka, Japan

**Keywords:** *Prdm13*, retina, amacrine cell, optokinetic response, aliasing

First, on behalf of all of the authors of our paper, we thank Drs. Bowrey and James for their interest in our paper and for giving us their comments on the OKRs (Optokinetic Responses) of *Prdm13*-deficient (*Prdm13*^−∕−^) mice (Watanabe et al., 2015).

Drs. Bowrey and James hypothesized that *Prdm13*^−∕−^ mice showed enhanced sensitivities to moving visual stimuli through “aliasing” caused by the decreased sampling function of the reduced numbers of amacrine cells in the retina (Bowrey and James, 2015). Aliasing is a phenomenon in which the presentation of continuously moving visual stimulus of high spatial frequency causes reduced neural sampling function, leading to misrecognition of a high frequency stimulus as a low frequency pattern (Gotz, 1964; Anderson and Hess, 1990; Coletta et al., 1990; Artal et al., 1995).

According to the previous studies on mouse visual function analysis, the optimal spatial frequency range, that which elicits smooth eye movement, is 0.01–0.5 cycle/degree, and the maximum spatial frequency for maintaining smooth eye movement without causing aliasing is 1.0 cycle/degree (Prusky et al., 2000; Geng et al., 2011). However, in fact, many experiments set the highest spatial frequencies lower than 1.0 cycle/degree (Prusky and Douglas, 2004; Prusky et al., 2004; van Alphen et al., 2010; Busse et al., 2011; Histed et al., 2012). In our study, we set the highest spatial frequency at 0.5 cycle/degree, at which aliasing is very unlikely to occur. Even if we suppose that aliasing can occur at 0.5 cycle/degree, the OKRs of *Prdm13*^−∕−^ mice at 0.5 cycle/degree were unchanged in both initial and late phases compared with those in WT mice. This strongly suggests that an aliasing effect, which shows stronger responses at higher frequencies, was not observed at 0.5 cycle/degree.

Visual responses in classical OKR were measured basically by whether or not the mouse head or eye moves. On the other hand, the visual responses used in our OKR system are based on the speed of the smooth eye movements elicited by moving visual stimuli. In other words, the classical OKR digitally detected the existence of responses to visual stimuli, whereas our OKR continuously measured the extent of moving stimuli speeds. Hence, if aliasing occurred in our OKR system, eye movement speed would become slower, and reduced OKRs would be observed.

Most studies of retinal sampling function have focused on photoreceptors and ganglion cells (Missotten, 1974; Thibos et al., 1987; Dacey, 1993). The relationship between amacrine cell subtypes and retinal sampling function has barely been explored. On the other hand, it has been reported that direction-selective ganglion cells (DSGCs) in the retina provide direct inputs to the brainstem structures involved in OKRs (Oyster et al., 1980; Yonehara et al., 2009; Kim et al., 2010; Kay et al., 2011). DSGC spike responses were elicited by moving grating stimuli at spatial frequencies of 0.025–0.2 cycles/degree and temporal frequencies of 0.25–5.33 cycles/s in the preferred direction (Hoggarth et al., 2015). These spatiotemporal tuning properties of DSGCs are similar to those of mouse OKRs (Tabata et al., 2010). In our study, *Prdm13*^−∕−^ mice showed OKRs at spatial frequencies of 0.03–0.25 cycles/degree and temporal frequencies of 0.375–12 cycles/s (Watanabe et al., 2015), which are consistent with the spatiotemporal frequency ranges of DSGCs. This suggests that DSGCs modulate the OKRs of *Prdm13*^−∕−^ mice, as we mentioned in the Discussion of our paper. Furthermore, Hoggarth suggested that the GABAergic wide-field amacrine cells modulate the spatiotemporal tuning properties of DSGCs (Hoggarth et al., 2015). Since a significant number of Prdm13-positive amacrine cells are GABAergic, GABAergic wide-field amacrine cells might be affected in *Prdm13*^−∕−^ mice. Hence, modulation of DSGCs may be the more probable mechanism affecting the OKRs of *Prdm13*^−∕−^ mice than aliasing. However, further elucidation of the functional mechanisms of Prdm13-positive amacrine cells in the retinal circuit is needed.

Taking the above considerations together, we conclude that OKR enhancement in *Prdm13*^−∕−^ mice is not due to aliasing. However, we do not deny the possibility that Prdm13-positive amacrine cells are involved in aliasing when it occurs. Further detailed analysis of *Prdm13*^−∕−^ mouse visual function will advance our understanding of information processing in the intricate retinal circuit.

## Author contributions

YS, SW, and TF wrote the response commentary.

## Funding

This work was supported by the Japan Science and Technology Agency (JST), Core Research for Evolutional Science and Technology (CREST), Hyogo Science and Techonology Association, The Osaka Community Foundation, and a Grant-in-Aid for Scientific Research (#15H04669).

### Conflict of interest statement

The authors declare that the research was conducted in the absence of any commercial or financial relationships that could be construed as a potential conflict of interest.
